# (2-{[2-(diphenyl­phosphino)phen­yl]thio}­phen­yl)diphenyl­phosphine sulfide

**DOI:** 10.1107/S1600536812041347

**Published:** 2012-10-10

**Authors:** Angel Alvarez-Larena, Francisco J. Martinez-Cuevas, Teresa Flor, Juli Real

**Affiliations:** a Servicio Difracción Rayos X, Universidad Autónoma de Barcelona, 08193 Cerdanyola, Spain; b Departamento Química, Universidad Autónoma de Barcelona, 08193 Cerdanyola, Spain

## Abstract

In the title compound, C_36_H_28_P_2_S_2_, the dihedral angle between the central benzene rings is 66.95 (13)°. In the crystal, molecules are linked *via* C_ar_—H⋯π and π–π inter­actions [shortest centroid–centroid distance between benzene rings = 3.897 (2) Å].

## Related literature

For related structures, see: Deb & Dutta (2010 ▶); Deb *et al.* (2010 ▶); Tooke *et al.* (2005 ▶); Pintado-Alba *et al.* (2004 ▶). For additional information on hemilabile ligands, see: Dallanegra *et al.* (2012 ▶); Marimuthu *et al.* (2012 ▶); Tello-López (2010 ▶).
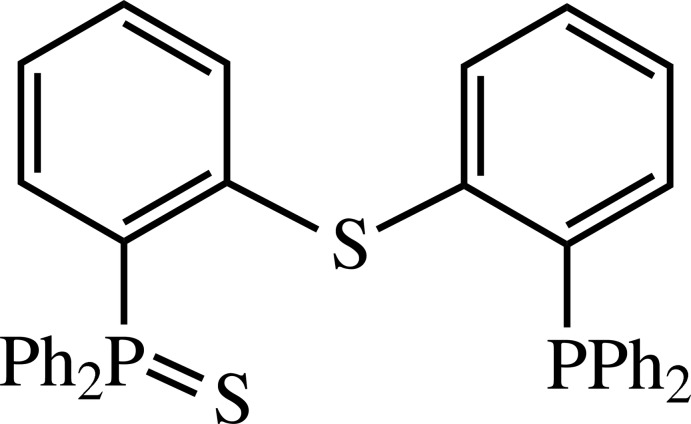


## Experimental

### 

#### Crystal data

C_36_H_28_P_2_S_2_
*M* *_r_* = 586.64
Triclinic, 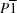

*a* = 10.8595 (10) Å
*b* = 11.0267 (10) Å
*c* = 13.3031 (12) Å
α = 76.404 (2)°
β = 79.151 (2)°
γ = 81.976 (2)°
*V* = 1513.1 (2) Å^3^
*Z* = 2
Mo *K*α radiation
μ = 0.31 mm^−1^
*T* = 296 K
0.34 × 0.22 × 0.20 mm

#### Data collection

Bruker SMART APEX diffractometer
Absorption correction: multi-scan (*SADABS*; Bruker, 2001 ▶) *T*_min_ = 0.806, *T*_max_ = 0.941
10362 measured reflections
6991 independent reflections
4414 reflections with *I* > 2σ(*I*)
*R*_int_ = 0.029

#### Refinement

*R*[*F* ^2^ > 2σ(*F* ^2^)] = 0.059
*wR*(*F* ^2^) = 0.135
*S* = 0.98
6991 reflections
361 parameters
H-atom parameters not refined
Δρ_max_ = 0.46 e Å^−3^
Δρ_min_ = −0.29 e Å^−3^

### 

Data collection: *SMART* (Bruker, 2001 ▶); cell refinement: *SAINT* (Bruker, 2001 ▶); data reduction: *SAINT*; program(s) used to solve structure: *SHELXTL* (Sheldrick, 2008 ▶); program(s) used to refine structure: *SHELXTL*; molecular graphics: *PLATON* (Spek, 2009 ▶); software used to prepare material for publication: *publCIF* (Westrip, 2010 ▶).

## Supplementary Material

Crystal structure: contains datablock(s) I, global. DOI: 10.1107/S1600536812041347/pk2445sup1.cif

Structure factors: contains datablock(s) I. DOI: 10.1107/S1600536812041347/pk2445Isup2.hkl

Supplementary material file. DOI: 10.1107/S1600536812041347/pk2445Isup3.cml

Additional supplementary materials:  crystallographic information; 3D view; checkCIF report

## Figures and Tables

**Table 1:** Hydrogen-bond geometry (Å, °) *Cg* is the centroid of the C41–C46 ring.

*D*—H⋯*A*	*D*—H	H⋯*A*	*D*⋯*A*	*D*—H⋯*A*
C23—H23⋯*Cg* ^i^	0.93	2.94	3.777 (4)	150

Symmetry code: (i) 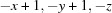.

**Fig. 1.**
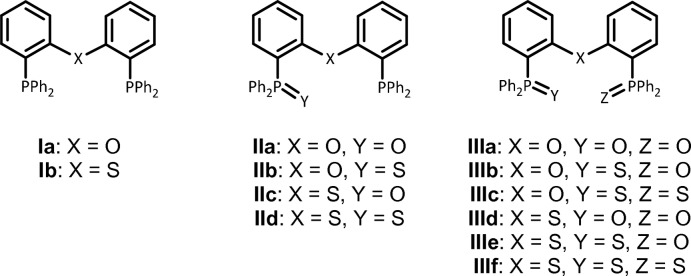
Examples of hemilabile ligands.

**Fig. 2.**
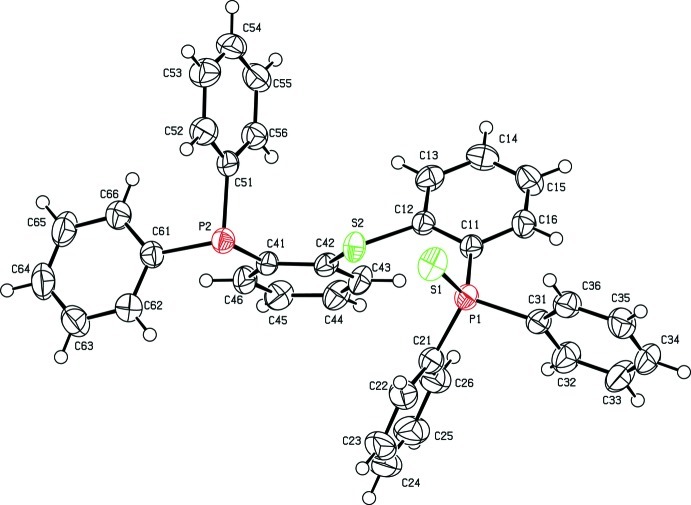
Molecular structure of the title compound with anisotropic displacement ellipsoids at the 50% probability level.

## supplementary crystallographic information

### Comment

Metal complexes of hemilabile ligands have been studied in the last few years due to their importance in homogeneous catalysis (Deb *et al.*, 2010; Dallanegra *et al.*, 2012; Marimuthu *et al.*, 2012). The phosphine derivatives (Ia,b), (IIa-d), and (IIIa-f) are a set of hemilabile ligands where the nature of the heteroatoms determines their coordinating ability towards soft or hard metals (Fig. 1). Crystal structures of (Ia), (IIa,b), and (IIIa) have been described (Pintado-Alba *et al.*, 2004; Tooke *et al.*, 2005; Deb *et al.*, 2010; Deb and Dutta, 2010). Here we present the structure of (IId). The molecular structure is shown in Fig. 2. The ability of this molecule to act as a ligand is related to its conformational freedom. The S(hinge)···S(arm) distance depends on the torsional angle C12—C11—P1═S1 whereas the S(arm)···P distance depends also on C11—C12—S2—C42 and C12—S2—C42—C41. In this way, the ligand can be coordinated to metals of different sizes. Values found in (IId) for the above torsional angles are -64.1 (2)°, -125.8 (2)° and -154.7 (2)° respectively. In this conformation, distances between coordinating positions are S1···S2 = 3.6803 (12) Å, S2···P2 = 3.1308 (11) Å, and S1···P2 = 6.0924 (13) Å.

### Experimental

Bis-[2-(diphenylphosphino)phenyl]sulfane (1.0 g, 1.8 mmol) (obtained as described by Tello-López, 2010), sulfur (58 mg, 1.8 mmol), and CH_2_Cl_2_ (50 ml) were placed in a 50 ml Schlenk flask. The mixture was stirred under nitrogen at room temperature for 48 h. The solution was evaporated to dryness and the solid was purified by silica gel chromatography (CH_2_Cl_2_/Hexane 3:1) and by crystallization from CH_2_Cl_2_/MeOH 1:3 to yield the title product (0.34 g, 32%). ^31^P{1H} NMR (101 MHz, CDCl_3_ 298 K): δ 43.1(*s*), -11.2(*s*) p.p.m. IR (KBr): 3052–2921, 1583–1477, 1434, 1100, 747, 692, 518 cm^-1^. Single crystals were obtained by evaporation of a toluene solution.

### Refinement

H atoms were positioned geometrically and refined using a riding model with C—H = 0.93 Å and with *U*_iso_(H) = 1.2 times *U*_eq_(C).

### Crystal data

**Table:**

C_36_H_28_P_2_S_2_	*Z* = 2
*M**_r_* = 586.64	*F*(000) = 612
Triclinic, *P*1	*D*_x_ = 1.288 Mg m^−^^3^
Hall symbol: -P 1	Mo *K*α radiation, λ = 0.71073 Å
*a* = 10.8595 (10) Å	Cell parameters from 1777 reflections
*b* = 11.0267 (10) Å	θ = 2.6–23.0°
*c* = 13.3031 (12) Å	µ = 0.31 mm^−^^1^
α = 76.404 (2)°	*T* = 296 K
β = 79.151 (2)°	Prism, colourless
γ = 81.976 (2)°	0.34 × 0.22 × 0.20 mm
*V* = 1513.1 (2) Å^3^	

### Data collection

**Table:**

Bruker SMART APEX diffractometer	6991 independent reflections
Radiation source: fine-focus sealed tube	4414 reflections with *I* > 2σ(*I*)
Graphite monochromator	*R*_int_ = 0.029
Detector resolution: 8.13 pixels mm^-1^	θ_max_ = 28.9°, θ_min_ = 1.6°
φ and ω scans	*h* = −14→14
Absorption correction: multi-scan (*SADABS*; Bruker, 2001)	*k* = −8→14
*T*_min_ = 0.806, *T*_max_ = 0.941	*l* = −17→17
10362 measured reflections	

### Refinement

**Table:**

Refinement on *F*^2^	Primary atom site location: structure-invariant direct methods
Least-squares matrix: full	Secondary atom site location: difference Fourier map
*R*[*F*^2^ > 2σ(*F*^2^)] = 0.059	Hydrogen site location: inferred from neighbouring sites
*wR*(*F*^2^) = 0.135	H-atom parameters not refined
*S* = 0.98	*w* = 1/[σ^2^(*F*_o_^2^) + (0.059*P*)^2^] where *P* = (*F*_o_^2^ + 2*F*_c_^2^)/3
6991 reflections	(Δ/σ)_max_ < 0.001
361 parameters	Δρ_max_ = 0.46 e Å^−^^3^
0 restraints	Δρ_min_ = −0.29 e Å^−^^3^

### Special details

**Table:**

Geometry. All e.s.d.'s (except the e.s.d. in the dihedral angle between two l.s. planes) are estimated using the full covariance matrix. The cell e.s.d.'s are taken into account individually in the estimation of e.s.d.'s in distances, angles and torsion angles; correlations between e.s.d.'s in cell parameters are only used when they are defined by crystal symmetry. An approximate (isotropic) treatment of cell e.s.d.'s is used for estimating e.s.d.'s involving l.s. planes.
Refinement. Refinement of *F*^2^ against ALL reflections. The weighted *R*-factor *wR* and goodness of fit *S* are based on *F*^2^, conventional *R*-factors *R* are based on *F*, with *F* set to zero for negative *F*^2^. The threshold expression of *F*^2^ > σ(*F*^2^) is used only for calculating *R*-factors(gt) *etc*. and is not relevant to the choice of reflections for refinement. *R*-factors based on *F*^2^ are statistically about twice as large as those based on *F*, and *R*- factors based on ALL data will be even larger.

### Fractional atomic coordinates and isotropic or equivalent isotropic displacement parameters (Å^2^)

**Table:**

	*x*	*y*	*z*	*U*_iso_*/*U*_eq_	
C41	0.4459 (2)	0.2347 (2)	0.3240 (2)	0.0322 (6)	
C42	0.3286 (3)	0.2950 (2)	0.3009 (2)	0.0337 (6)	
C43	0.2504 (3)	0.2351 (3)	0.2616 (2)	0.0421 (7)	
H43	0.1724	0.2752	0.2473	0.050*	
C44	0.2877 (3)	0.1157 (3)	0.2436 (2)	0.0452 (7)	
H44	0.2343	0.0758	0.2179	0.054*	
C45	0.4029 (3)	0.0558 (3)	0.2634 (2)	0.0466 (8)	
H45	0.4281	−0.0240	0.2506	0.056*	
C46	0.4811 (3)	0.1158 (3)	0.3026 (2)	0.0426 (7)	
H46	0.5596	0.0754	0.3150	0.051*	
C51	0.4753 (2)	0.2811 (3)	0.5186 (2)	0.0361 (6)	
C52	0.5194 (3)	0.3387 (3)	0.5850 (2)	0.0468 (7)	
H52	0.5817	0.3929	0.5580	0.056*	
C53	0.4717 (3)	0.3165 (3)	0.6914 (3)	0.0547 (9)	
H53	0.5032	0.3543	0.7355	0.066*	
C54	0.3779 (3)	0.2387 (3)	0.7314 (2)	0.0543 (9)	
H54	0.3461	0.2233	0.8027	0.065*	
C55	0.3308 (3)	0.1834 (3)	0.6661 (2)	0.0507 (8)	
H55	0.2659	0.1322	0.6929	0.061*	
C56	0.3800 (3)	0.2038 (3)	0.5604 (2)	0.0423 (7)	
H56	0.3486	0.1651	0.5169	0.051*	
C61	0.6912 (2)	0.2077 (3)	0.3795 (2)	0.0382 (7)	
C62	0.7832 (3)	0.2262 (3)	0.2904 (2)	0.0507 (8)	
H62	0.7688	0.2910	0.2337	0.061*	
C63	0.8948 (3)	0.1504 (4)	0.2849 (3)	0.0626 (10)	
H63	0.9544	0.1632	0.2243	0.075*	
C64	0.9187 (3)	0.0555 (4)	0.3690 (3)	0.0650 (11)	
H64	0.9944	0.0042	0.3654	0.078*	
C65	0.8313 (3)	0.0372 (3)	0.4570 (3)	0.0601 (9)	
H65	0.8479	−0.0264	0.5140	0.072*	
C66	0.7179 (3)	0.1118 (3)	0.4634 (2)	0.0479 (8)	
H66	0.6590	0.0977	0.5244	0.057*	
C11	0.0501 (2)	0.5606 (2)	0.2821 (2)	0.0330 (6)	
C12	0.1193 (2)	0.4622 (2)	0.3423 (2)	0.0330 (6)	
C13	0.0563 (3)	0.3762 (3)	0.4220 (2)	0.0423 (7)	
H13	0.1023	0.3118	0.4623	0.051*	
C14	−0.0737 (3)	0.3851 (3)	0.4424 (2)	0.0474 (8)	
H14	−0.1148	0.3265	0.4957	0.057*	
C15	−0.1419 (3)	0.4801 (3)	0.3838 (2)	0.0506 (8)	
H15	−0.2295	0.4859	0.3970	0.061*	
C16	−0.0809 (3)	0.5676 (3)	0.3054 (2)	0.0429 (7)	
H16	−0.1284	0.6327	0.2671	0.051*	
C21	0.2330 (3)	0.6318 (3)	0.0877 (2)	0.0423 (7)	
C22	0.3334 (3)	0.7007 (3)	0.0351 (3)	0.0560 (9)	
H22	0.3477	0.7705	0.0576	0.067*	
C23	0.4119 (3)	0.6655 (4)	−0.0504 (3)	0.0701 (11)	
H23	0.4779	0.7125	−0.0856	0.084*	
C24	0.3926 (4)	0.5619 (4)	−0.0831 (3)	0.0733 (12)	
H24	0.4457	0.5386	−0.1404	0.088*	
C25	0.2949 (4)	0.4923 (4)	−0.0316 (3)	0.0667 (10)	
H25	0.2823	0.4217	−0.0538	0.080*	
C26	0.2147 (3)	0.5276 (3)	0.0538 (2)	0.0540 (9)	
H26	0.1484	0.4806	0.0882	0.065*	
C31	−0.0005 (3)	0.7811 (3)	0.1210 (2)	0.0383 (7)	
C32	−0.0465 (3)	0.7388 (3)	0.0465 (2)	0.0541 (8)	
H32	−0.0143	0.6609	0.0321	0.065*	
C33	−0.1383 (3)	0.8088 (3)	−0.0065 (3)	0.0614 (10)	
H33	−0.1678	0.7785	−0.0561	0.074*	
C34	−0.1865 (3)	0.9242 (3)	0.0142 (3)	0.0579 (9)	
H34	−0.2482	0.9723	−0.0221	0.070*	
C35	−0.1440 (3)	0.9685 (3)	0.0880 (3)	0.0552 (9)	
H35	−0.1771	1.0461	0.1024	0.066*	
C36	−0.0508 (3)	0.8964 (3)	0.1412 (2)	0.0448 (7)	
H36	−0.0220	0.9266	0.1913	0.054*	
P1	0.12398 (7)	0.69086 (7)	0.19072 (6)	0.03650 (19)	
P2	0.54595 (7)	0.31492 (7)	0.37954 (6)	0.03589 (19)	
S1	0.19859 (8)	0.79139 (8)	0.26148 (7)	0.0525 (2)	
S2	0.28748 (7)	0.45079 (7)	0.32212 (6)	0.03975 (19)	

### Atomic displacement parameters (Å^2^)

**Table:**

	*U*^11^	*U*^22^	*U*^33^	*U*^12^	*U*^13^	*U*^23^
C41	0.0324 (14)	0.0306 (14)	0.0319 (14)	0.0022 (11)	−0.0072 (11)	−0.0047 (12)
C42	0.0402 (15)	0.0285 (14)	0.0313 (14)	0.0029 (12)	−0.0089 (12)	−0.0055 (12)
C43	0.0443 (17)	0.0390 (17)	0.0448 (17)	0.0046 (14)	−0.0201 (14)	−0.0077 (14)
C44	0.0531 (19)	0.0401 (17)	0.0475 (17)	−0.0036 (14)	−0.0196 (15)	−0.0107 (15)
C45	0.058 (2)	0.0345 (16)	0.0486 (18)	0.0053 (15)	−0.0144 (15)	−0.0131 (15)
C46	0.0408 (17)	0.0408 (17)	0.0448 (17)	0.0091 (13)	−0.0115 (14)	−0.0105 (14)
C51	0.0328 (15)	0.0362 (15)	0.0379 (15)	0.0059 (12)	−0.0083 (12)	−0.0083 (13)
C52	0.0452 (18)	0.0511 (19)	0.0460 (18)	−0.0071 (15)	−0.0071 (14)	−0.0131 (16)
C53	0.062 (2)	0.061 (2)	0.0471 (19)	−0.0007 (18)	−0.0130 (17)	−0.0214 (18)
C54	0.056 (2)	0.059 (2)	0.0393 (17)	0.0110 (17)	0.0005 (16)	−0.0110 (17)
C55	0.0415 (18)	0.051 (2)	0.0509 (19)	−0.0021 (15)	0.0034 (15)	−0.0045 (16)
C56	0.0410 (17)	0.0403 (17)	0.0439 (17)	−0.0020 (13)	−0.0065 (14)	−0.0069 (14)
C61	0.0321 (15)	0.0417 (17)	0.0422 (16)	−0.0028 (13)	−0.0077 (13)	−0.0108 (14)
C62	0.0440 (18)	0.061 (2)	0.0461 (18)	−0.0004 (16)	−0.0088 (15)	−0.0119 (17)
C63	0.0427 (19)	0.088 (3)	0.062 (2)	0.0040 (19)	−0.0042 (17)	−0.034 (2)
C64	0.043 (2)	0.083 (3)	0.079 (3)	0.0206 (19)	−0.0251 (19)	−0.041 (2)
C65	0.056 (2)	0.060 (2)	0.067 (2)	0.0154 (18)	−0.0290 (19)	−0.0149 (19)
C66	0.0417 (17)	0.0490 (19)	0.0471 (18)	0.0038 (15)	−0.0081 (14)	−0.0028 (16)
C11	0.0327 (14)	0.0326 (15)	0.0331 (14)	−0.0001 (12)	−0.0066 (12)	−0.0069 (12)
C12	0.0338 (14)	0.0332 (15)	0.0328 (14)	0.0000 (12)	−0.0077 (12)	−0.0088 (12)
C13	0.0536 (19)	0.0327 (16)	0.0380 (16)	−0.0019 (14)	−0.0106 (14)	−0.0015 (13)
C14	0.055 (2)	0.0422 (18)	0.0417 (17)	−0.0144 (15)	0.0027 (15)	−0.0047 (15)
C15	0.0382 (17)	0.0511 (19)	0.057 (2)	−0.0060 (15)	0.0022 (15)	−0.0084 (17)
C16	0.0370 (16)	0.0411 (17)	0.0465 (17)	0.0038 (13)	−0.0078 (13)	−0.0051 (15)
C21	0.0421 (17)	0.0389 (17)	0.0392 (16)	0.0046 (13)	−0.0072 (13)	0.0003 (14)
C22	0.0467 (19)	0.056 (2)	0.053 (2)	−0.0013 (16)	−0.0026 (16)	0.0043 (17)
C23	0.054 (2)	0.080 (3)	0.051 (2)	0.006 (2)	0.0097 (17)	0.015 (2)
C24	0.076 (3)	0.078 (3)	0.045 (2)	0.024 (2)	0.0067 (19)	−0.003 (2)
C25	0.083 (3)	0.062 (2)	0.049 (2)	0.014 (2)	−0.004 (2)	−0.0173 (19)
C26	0.058 (2)	0.050 (2)	0.0473 (19)	0.0020 (16)	−0.0004 (16)	−0.0087 (17)
C31	0.0409 (16)	0.0348 (16)	0.0342 (15)	0.0020 (13)	−0.0061 (13)	−0.0010 (13)
C32	0.062 (2)	0.0462 (19)	0.0534 (19)	0.0093 (16)	−0.0195 (17)	−0.0096 (17)
C33	0.069 (2)	0.066 (2)	0.050 (2)	0.0031 (19)	−0.0292 (18)	−0.0048 (18)
C34	0.053 (2)	0.060 (2)	0.050 (2)	0.0073 (17)	−0.0192 (16)	0.0099 (18)
C35	0.051 (2)	0.0460 (19)	0.056 (2)	0.0128 (16)	−0.0047 (16)	−0.0009 (17)
C36	0.0499 (18)	0.0389 (17)	0.0403 (16)	0.0025 (14)	−0.0054 (14)	−0.0038 (14)
P1	0.0373 (4)	0.0316 (4)	0.0373 (4)	0.0013 (3)	−0.0079 (3)	−0.0023 (3)
P2	0.0335 (4)	0.0352 (4)	0.0375 (4)	−0.0007 (3)	−0.0076 (3)	−0.0052 (3)
S1	0.0574 (5)	0.0426 (5)	0.0608 (5)	−0.0065 (4)	−0.0208 (4)	−0.0079 (4)
S2	0.0366 (4)	0.0311 (4)	0.0525 (4)	0.0034 (3)	−0.0158 (3)	−0.0080 (3)

### Geometric parameters (Å, º)

**Table:**

C41—C46	1.394 (4)	C11—C12	1.405 (4)
C41—C42	1.405 (3)	C11—P1	1.822 (3)
C41—P2	1.840 (3)	C12—C13	1.388 (4)
C42—C43	1.385 (4)	C12—S2	1.786 (3)
C42—S2	1.788 (3)	C13—C14	1.381 (4)
C43—C44	1.384 (4)	C13—H13	0.9300
C43—H43	0.9300	C14—C15	1.368 (4)
C44—C45	1.372 (4)	C14—H14	0.9300
C44—H44	0.9300	C15—C16	1.380 (4)
C45—C46	1.384 (4)	C15—H15	0.9300
C45—H45	0.9300	C16—H16	0.9300
C46—H46	0.9300	C21—C26	1.381 (4)
C51—C56	1.383 (4)	C21—C22	1.397 (4)
C51—C52	1.386 (4)	C21—P1	1.815 (3)
C51—P2	1.836 (3)	C22—C23	1.385 (5)
C52—C53	1.388 (4)	C22—H22	0.9300
C52—H52	0.9300	C23—C24	1.369 (5)
C53—C54	1.372 (4)	C23—H23	0.9300
C53—H53	0.9300	C24—C25	1.375 (5)
C54—C55	1.375 (4)	C24—H24	0.9300
C54—H54	0.9300	C25—C26	1.393 (4)
C55—C56	1.383 (4)	C25—H25	0.9300
C55—H55	0.9300	C26—H26	0.9300
C56—H56	0.9300	C31—C36	1.379 (4)
C61—C66	1.389 (4)	C31—C32	1.384 (4)
C61—C62	1.393 (4)	C31—P1	1.828 (3)
C61—P2	1.834 (3)	C32—C33	1.371 (4)
C62—C63	1.373 (4)	C32—H32	0.9300
C62—H62	0.9300	C33—C34	1.376 (4)
C63—C64	1.377 (5)	C33—H33	0.9300
C63—H63	0.9300	C34—C35	1.369 (4)
C64—C65	1.356 (5)	C34—H34	0.9300
C64—H64	0.9300	C35—C36	1.392 (4)
C65—C66	1.383 (4)	C35—H35	0.9300
C65—H65	0.9300	C36—H36	0.9300
C66—H66	0.9300	P1—S1	1.9494 (11)
C11—C16	1.393 (4)		
			
S1···S2	3.6803 (12)	S2···P2	3.1308 (11)
			
C46—C41—C42	117.6 (2)	C11—C12—S2	120.9 (2)
C46—C41—P2	123.1 (2)	C14—C13—C12	120.9 (3)
C42—C41—P2	119.37 (19)	C14—C13—H13	119.5
C43—C42—C41	120.3 (2)	C12—C13—H13	119.5
C43—C42—S2	122.3 (2)	C15—C14—C13	119.8 (3)
C41—C42—S2	117.4 (2)	C15—C14—H14	120.1
C44—C43—C42	120.3 (3)	C13—C14—H14	120.1
C44—C43—H43	119.9	C14—C15—C16	120.1 (3)
C42—C43—H43	119.9	C14—C15—H15	120.0
C45—C44—C43	120.6 (3)	C16—C15—H15	120.0
C45—C44—H44	119.7	C15—C16—C11	121.6 (3)
C43—C44—H44	119.7	C15—C16—H16	119.2
C44—C45—C46	119.1 (3)	C11—C16—H16	119.2
C44—C45—H45	120.4	C26—C21—C22	118.7 (3)
C46—C45—H45	120.4	C26—C21—P1	122.6 (2)
C45—C46—C41	122.1 (3)	C22—C21—P1	118.4 (2)
C45—C46—H46	119.0	C23—C22—C21	120.3 (4)
C41—C46—H46	119.0	C23—C22—H22	119.8
C56—C51—C52	118.3 (3)	C21—C22—H22	119.8
C56—C51—P2	124.2 (2)	C24—C23—C22	120.3 (4)
C52—C51—P2	117.5 (2)	C24—C23—H23	119.9
C51—C52—C53	120.9 (3)	C22—C23—H23	119.9
C51—C52—H52	119.6	C23—C24—C25	120.2 (3)
C53—C52—H52	119.6	C23—C24—H24	119.9
C54—C53—C52	119.8 (3)	C25—C24—H24	119.9
C54—C53—H53	120.1	C24—C25—C26	120.0 (4)
C52—C53—H53	120.1	C24—C25—H25	120.0
C53—C54—C55	120.0 (3)	C26—C25—H25	120.0
C53—C54—H54	120.0	C21—C26—C25	120.5 (3)
C55—C54—H54	120.0	C21—C26—H26	119.8
C54—C55—C56	120.1 (3)	C25—C26—H26	119.8
C54—C55—H55	120.0	C36—C31—C32	117.8 (3)
C56—C55—H55	120.0	C36—C31—P1	119.8 (2)
C55—C56—C51	120.9 (3)	C32—C31—P1	122.5 (2)
C55—C56—H56	119.6	C33—C32—C31	121.7 (3)
C51—C56—H56	119.6	C33—C32—H32	119.2
C66—C61—C62	117.5 (3)	C31—C32—H32	119.2
C66—C61—P2	124.6 (2)	C32—C33—C34	119.7 (3)
C62—C61—P2	117.9 (2)	C32—C33—H33	120.2
C63—C62—C61	121.2 (3)	C34—C33—H33	120.2
C63—C62—H62	119.4	C35—C34—C33	120.2 (3)
C61—C62—H62	119.4	C35—C34—H34	119.9
C62—C63—C64	120.2 (3)	C33—C34—H34	119.9
C62—C63—H63	119.9	C34—C35—C36	119.5 (3)
C64—C63—H63	119.9	C34—C35—H35	120.2
C65—C64—C63	119.6 (3)	C36—C35—H35	120.2
C65—C64—H64	120.2	C31—C36—C35	121.2 (3)
C63—C64—H64	120.2	C31—C36—H36	119.4
C64—C65—C66	120.9 (3)	C35—C36—H36	119.4
C64—C65—H65	119.5	C21—P1—C11	109.24 (13)
C66—C65—H65	119.5	C21—P1—C31	102.63 (13)
C65—C66—C61	120.6 (3)	C11—P1—C31	105.41 (13)
C65—C66—H66	119.7	C21—P1—S1	114.07 (11)
C61—C66—H66	119.7	C11—P1—S1	112.23 (9)
C16—C11—C12	117.9 (3)	C31—P1—S1	112.52 (10)
C16—C11—P1	119.0 (2)	C61—P2—C51	101.84 (12)
C12—C11—P1	122.4 (2)	C61—P2—C41	101.92 (12)
C13—C12—C11	119.7 (3)	C51—P2—C41	100.97 (13)
C13—C12—S2	119.4 (2)	C12—S2—C42	101.92 (13)
			
C46—C41—C42—C43	2.1 (4)	P1—C21—C26—C25	174.3 (3)
P2—C41—C42—C43	−178.3 (2)	C24—C25—C26—C21	−0.4 (5)
C46—C41—C42—S2	−176.8 (2)	C36—C31—C32—C33	0.5 (5)
P2—C41—C42—S2	2.7 (3)	P1—C31—C32—C33	−178.8 (3)
C41—C42—C43—C44	−0.8 (4)	C31—C32—C33—C34	0.0 (5)
S2—C42—C43—C44	178.1 (2)	C32—C33—C34—C35	−0.6 (5)
C42—C43—C44—C45	−0.6 (4)	C33—C34—C35—C36	0.6 (5)
C43—C44—C45—C46	0.6 (5)	C32—C31—C36—C35	−0.6 (4)
C44—C45—C46—C41	0.9 (5)	P1—C31—C36—C35	178.8 (2)
C42—C41—C46—C45	−2.2 (4)	C34—C35—C36—C31	0.0 (5)
P2—C41—C46—C45	178.3 (2)	C26—C21—P1—C11	30.5 (3)
C56—C51—C52—C53	1.9 (4)	C22—C21—P1—C11	−154.9 (2)
P2—C51—C52—C53	−179.2 (2)	C26—C21—P1—C31	−81.0 (3)
C51—C52—C53—C54	−1.4 (5)	C22—C21—P1—C31	93.6 (3)
C52—C53—C54—C55	−0.4 (5)	C26—C21—P1—S1	157.0 (2)
C53—C54—C55—C56	1.5 (5)	C22—C21—P1—S1	−28.4 (3)
C54—C55—C56—C51	−1.0 (5)	C16—C11—P1—C21	−126.7 (2)
C52—C51—C56—C55	−0.7 (4)	C12—C11—P1—C21	63.4 (2)
P2—C51—C56—C55	−179.6 (2)	C16—C11—P1—C31	−17.1 (2)
C66—C61—C62—C63	1.6 (5)	C12—C11—P1—C31	173.0 (2)
P2—C61—C62—C63	179.5 (2)	C16—C11—P1—S1	105.7 (2)
C61—C62—C63—C64	−1.2 (5)	C12—C11—P1—S1	−64.1 (2)
C62—C63—C64—C65	0.1 (5)	C36—C31—P1—C21	−137.5 (2)
C63—C64—C65—C66	0.6 (6)	C32—C31—P1—C21	41.8 (3)
C64—C65—C66—C61	−0.2 (5)	C36—C31—P1—C11	108.2 (2)
C62—C61—C66—C65	−0.8 (5)	C32—C31—P1—C11	−72.5 (3)
P2—C61—C66—C65	−178.6 (2)	C36—C31—P1—S1	−14.4 (3)
C16—C11—C12—C13	0.0 (4)	C32—C31—P1—S1	164.9 (2)
P1—C11—C12—C13	170.0 (2)	C66—C61—P2—C51	11.4 (3)
C16—C11—C12—S2	−176.8 (2)	C62—C61—P2—C51	−166.4 (2)
P1—C11—C12—S2	−6.9 (3)	C66—C61—P2—C41	−92.7 (3)
C11—C12—C13—C14	0.7 (4)	C62—C61—P2—C41	89.6 (3)
S2—C12—C13—C14	177.6 (2)	C56—C51—P2—C61	−99.5 (2)
C12—C13—C14—C15	−0.4 (4)	C52—C51—P2—C61	81.6 (2)
C13—C14—C15—C16	−0.6 (5)	C56—C51—P2—C41	5.3 (3)
C14—C15—C16—C11	1.3 (5)	C52—C51—P2—C41	−173.6 (2)
C12—C11—C16—C15	−1.0 (4)	C46—C41—P2—C61	6.1 (3)
P1—C11—C16—C15	−171.3 (2)	C42—C41—P2—C61	−173.4 (2)
C26—C21—C22—C23	0.9 (5)	C46—C41—P2—C51	−98.7 (2)
P1—C21—C22—C23	−173.9 (3)	C42—C41—P2—C51	81.9 (2)
C21—C22—C23—C24	−0.9 (5)	C13—C12—S2—C42	57.4 (2)
C22—C23—C24—C25	0.2 (6)	C11—C12—S2—C42	−125.8 (2)
C23—C24—C25—C26	0.4 (6)	C43—C42—S2—C12	26.4 (3)
C22—C21—C26—C25	−0.2 (5)	C41—C42—S2—C12	−154.7 (2)

### Hydrogen-bond geometry (Å, º)

Cg is the centroid of the C41–C46 ring.

**Table:**

*D*—H···*A*	*D*—H	H···*A*	*D*···*A*	*D*—H···*A*
C23—H23···*Cg*^i^	0.93	2.94	3.777 (4)	150

Symmetry code: (i) −*x*+1, −*y*+1, −*z*.
